# Optimizing PCF-SPR sensor design through Taguchi approach, machine learning, and genetic algorithms

**DOI:** 10.1038/s41598-024-55817-9

**Published:** 2024-04-03

**Authors:** Sameh Kaziz, Fraj Echouchene, Mohamed Hichem Gazzah

**Affiliations:** 1 NANOMISENE Laboratory, LR16CRMN01, Centre for Research on Microelectronics and Nanotechnology (CRMN) of Sousse Technopole, Sahloul, B.P.334, 4054 Sousse, Tunisia; 2 Electronic and Microelectronics Lab, Department of Physics, Faculty of Science of Monastir, University of Monastir, 5019 Monastir, Tunisia; 3 Quantum and Statistical Physics Laboratory, Faculty of Sciences of Monastir, University of Monastir, Environment Boulevard, 5019 Monastir, Tunisia

**Keywords:** Genetic algorithm, Surface plasmon resonance, Photonic crystal fiber, Multi-layer perceptron, Particle swarm optimization, Taguchi approach, Optics and photonics, Optical physics

## Abstract

Designing Photonic Crystal Fibers incorporating the Surface Plasmon Resonance Phenomenon (PCF-SPR) has led to numerous interesting applications. This investigation presents an exceptionally responsive surface plasmon resonance sensor, seamlessly integrated into a dual-core photonic crystal fiber, specifically designed for low refractive index (RI) detection. The integration of a plasmonic material, namely silver (Ag), externally deposited on the fiber structure, facilitates real-time monitoring of variations in the refractive index of the surrounding medium. To ensure long-term functionality and prevent oxidation, a thin layer of titanium dioxide (TiO_2_) covers the silver coating. To optimize the sensor, five key design parameters, including pitch, air hole diameter, and silver thickness, are fine-tuned using the Taguchi L_8_(2^5^) orthogonal array. The optimal results obtained present spectral and amplitude sensitivities that reach remarkable values of 10,000 nm/RIU and 235,882 RIU-1, respectively. In addition, Artificial Neural Network (ANN) optimization techniques, specifically Multi-Layer Perceptron (MLP) and Particle Swarm Optimization (PSO), are used to predict a critical optical property of the sensor confinement loss (α_loss_). These predictions are derived from the same input structure parameters that are present in the full L_32_(2^5^) design experiment. A genetic algorithm (GA) is then applied for optimization with the goal of maximizing the confinement loss. Our results highlight the effectiveness of training PSO artificial neural networks and demonstrate their ability to quickly and accurately predict results for unknown geometric dimensions, demonstrating their significant potential in this innovative context. The proposed sensor design can be used for various applications including pharmaceutical inspection and detection of low refractive index analytes.

## Introduction

The optical phenomenon known as surface plasmon resonance (SPR) occurs when free electrons oscillate at the interface between a metallic surface and a dielectric layer. In this fascinating phenomenon, the photon wavelengths of the incident electromagnetic wave align with the wavelengths of the surface electrons, especially under p-polarized light radiation^1^. This unique phenomenon has spurred extensive research into SPR sensors, primarily because of their attractive properties. These sensors offer efficiency, precision in sensing, fast response times, real-time and label-free detection, and an exceptional ability to effectively control light^2^. Traditional SPR sensors have been designed using prisms, fiber Bragg gratings, slot waveguides, and V-groove waveguides. However, these designs tend to be bulky and costly^2^. To overcome these limitations, SPR sensors based on photonic crystal fibers (PCFs) have been introduced. PCF-based sensors provide portability, compactness, and the ability for remote sensing. Various PCF-SPR structures have been investigated for different sensing applications. These include configurations such as microfluidic slot-based designs, external metal-coated structures, long-period fiber Bragg gratings, internal metal-coated structures, and D-shaped structures, among others^2^. A PCF-SPR sensor uses two different sensing configurations: external and internal. In the internal sensing approach, the analyte selectively occupies the air holes in the fiber. This mechanism enhances sensitivity because the introduced analyte directly modifies the initial refractive index distribution of the fiber. However, internal sensing is not suitable for real-time and distributed sensing applications due to its impracticality and susceptibility to significant propagation losses. To overcome these challenges, the external sensing technique is used. In this method, the analyte is located on the surface of the PCF, eliminating the need for analyte infiltration into the fiber. The external sensing technique has gained popularity due to its ease of detection and practical implementation^3^. In previous research, a gold lattice PCF-SPR sensor was introduced that achieved an impressive wavelength sensitivity (Ws) of 3340 nm/RIU^4^. Another study reported a D-shaped PCF sensor with a sensing range of 1.33 to 1.43, resulting in a maximum Ws of 46,000 nm/RIU^5^.

In a separate study, a gold-plated D-shaped PCF-SPR sensor with a refractive index (RI) detection range of 1.33 to 1.38 and a maximum sensitivity of 10,493 nm/RIU at an RI of 1.38 was discussed^6^. Numerous other PCF-based SPR sensors capable of detecting analytes with RI values as low as 1.33 have been documented in various studies^4–14^. However, most of this work has focused primarily on sensor structures suitable for analytes with RI values greater than 1.33. Research on PCF-SPR sensors capable of detecting lower RIs, particularly those below 1.30, has been relatively limited^3^. The current landscape demands sensors capable of detecting low RIs as applications emerge in diverse fields, including aerogels^15^, halogenated ethers^16^, sevoflurane, pharmaceuticals, and more. Recognizing this need, a few PCF-SPR sensors have emerged to address low-RI analyte detection^16–21^, with different WS values, including 13,500, 6000, 11,055, 20,000, 13,000, and 51,000 nm/RIU, respectively. However, it's noteworthy that only two of these studies^19,21^ reported the assessment of amplification sensitivity (As), with values of approximately 1054 and 1872 RIU-1, respectively. This underscores the untapped potential for PCF-SPR sensors capable of detecting lower refractive indices with improved sensitivity in both interrogation methods.

In this research, we have introduced and performed a comprehensive numerical analysis of a dual-core photonic crystal fiber surface plasmon resonance (PCF-SPR) sensor specifically designed for low refractive index detection. The improved performance of the sensor is achieved by incorporating a dual sensing channel created by a microchannel and a bimetallic configuration^22^. This innovative design improves the sensitivity of the sensor in both wavelength and amplitude interrogation methods. The addition of a titanium dioxide (TiO2) layer on top of the silver coating plays a key role in improving sensor performance. It generates a significant number of surface electrons that effectively attract the field from the core, resulting in a robust interaction with the plasmonic mode.

Accurate modeling and optimization of photonic crystal structures typically depends on numerical methods, including the finite difference method^23^, the finite element method (FEM)^24^, the block-iterative frequency domain method^25^, and the plane wave expansion method^26,27^.

However, it's worth noting that these methods require significant computational resources, especially when faced with complex photonic crystal structures that require multiple simulations to achieve an optimized design. Moreover, the computational burden of these iterative analyses is directly influenced by the number of input design parameters to be optimized. Therefore, in our study, we used the Taguchi approach to optimize five critical structural parameters of the PCF sensor. These parameters include pitch, air hole diameter, and silver layer thickness. By using the Taguchi approach, we were able to streamline the optimization process and achieve our goals with a limited number of simulations^28–30^.

Recently, the field of machine learning (ML) and deep learning has emerged as a dominant force in various fields, including computer vision, robotics, chatbots, natural language processing, and many others. In addition, researchers have expanded their exploration of the applicability of machine learning to the field of photonics. This expansion has included diverse areas such as multimode fibers^31^, plasmonics^32^, biosensing^33^, and metamaterials^34^ and networking^35^. In one notable case, Kiarashinejad et al.^36^ introduced a deep learning-based algorithm that used dimensionality reduction techniques to gain insight into the interactions between electromagnetic waves and nanostructures. In addition, a geometric deep learning approach has been used to study nanophotonic structures^37^. In 2018, the integration of extreme learning machines and deep learning techniques has been used to compute dispersion relations^38^ and optimize Q factors^39^ for photonic crystals.

A genetic algorithm is a search and optimization method inspired by natural selection and genetics. It is used to solve complex problems by evolving a population of potential solutions over generations. Through operations such as reproduction, mutation, and selection, genetic algorithms aim to obtain increasingly better solutions over time, simulating the process of biological evolution to find optimal or near-optimal solutions. These algorithms are widely used in optimization and heuristic search.

In our work, we aim to harness the innovative synergy of Taguchi methodology and artificial intelligence, leveraging machine learning techniques to forecast confinement losses in photonic crystal fibers. We combine finite element simulations with artificial neural networks (ANN) to facilitate fast and accurate computations. The motivation of this work revolves around the design of a simple feed-forward Multilayer Perceptron (MLP) and Particle Swarm Optimization (PSO) models that can be trained to estimate critical parameters such as confinement loss (α_loss_) for a PCF structure. Furthermore, Genetic Algorithm (GA) is applied for optimization to maximize the confinement loss in the sensor.

## Design and numerical simulation

**Figure 1:**
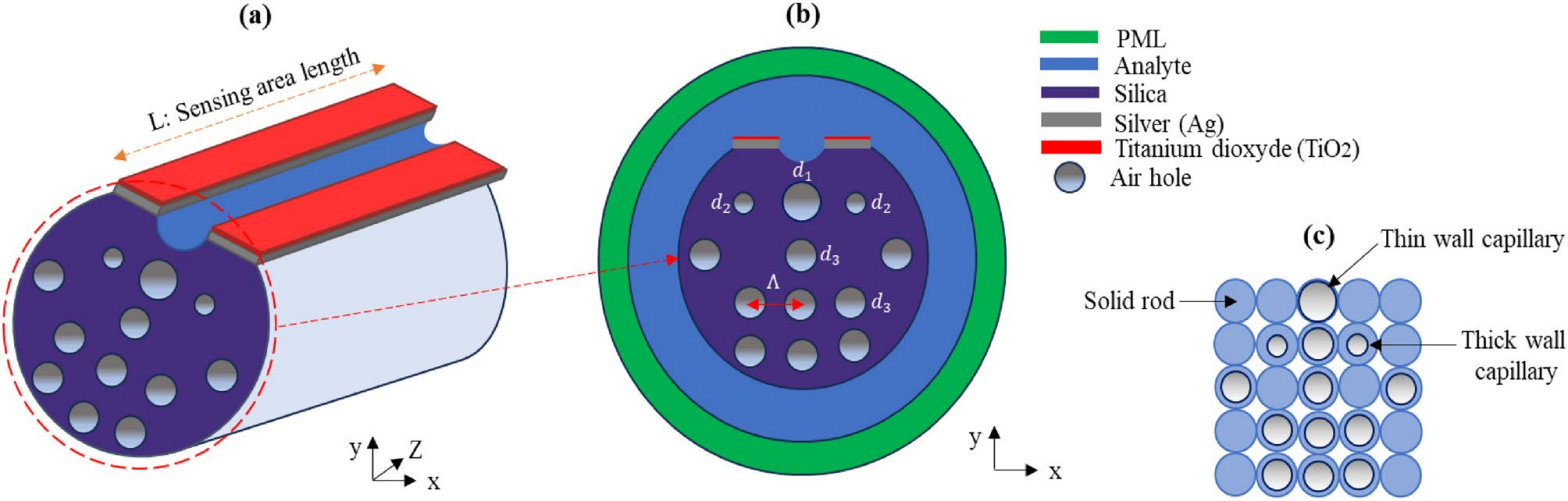
Illustration depicting the proposed Dual-core PCF sensor configuration (**a**), the x–y cross-sectional view (**b**), and the stacked preform of the fiber (**c**).

The proposed dual-core PCF sensor configuration and x–y cross-sectional view are shown in Fig. 1a and b. This novel sensor design is organized in a square lattice with two layers of air holes (Fig. 1c). The sensing area spans a length of L = 1 mm. To improve the interaction between the core-guided and surface plasmon polariton (SPP) modes, we reduced the size of two air holes (d_2_) located at the top of the initial ring. In addition, we excluded two air holes located in the center of the initial ring when fabricating the dual-core structure.

The manufacturing process involves the layering of capillaries and solid rods, followed by drawing at a certain speed to form the fiber. Different dimensions of air holes, including both large and small sizes, and absence of air holes are achieved by using thin and thick capillaries and solid rods, respectively^9^. Upon completion of fiber fabrication, a polishing technique is implemented^5^. This technique involves polishing a segment of the fiber, incorporating the large thin-walled capillary from the second ring, while the remaining part of the capillary forms the microchannel. Finally, a chemical deposition technique^18,19^ is used to deposit a coating of silver and TiO_2_ on the polished side of the fiber.

The finite element method (FEM) was used for the numerical analysis of the proposed sensor. In order to improve the absorption of the radiation power, a perfectly matched layer was included as the outermost layer. To achieve the highest simulation accuracy, a very fine mesh element was used. The optimized structural parameters consist of the diameters of the air holes (d_1_, d_2_, d_3_), the pitch (Λ), and the thickness of the silver layer (t_Ag_). Furthermore, the opening of the microchannel is set to 1.75 µm.

The dielectric constant of silver is determined using the Drude model, as described in the reference^40^:

1 $${\varepsilon }_{Ag}\left(\omega \right)={\varepsilon }_{\infty }-\frac{{\omega }_{p}^{2}}{\omega (\omega +i{\omega }_{\tau })}$$

where ${\varepsilon }_{\infty }=9.84$, is the dimensionless high-frequency (infinite frequency) permittivity, ${\omega }_{p}=1.367\times {10}^{16} rad/s$, is the plasma frequency, and ${\omega }_{\tau }=1.018\times {10}^{14} rad/s$, is the collision frequency.

As for the background material, SiO_2_ is used, and its refractive index is determined using the following Sellmeier equation, as described in reference^41^.

2 $${n}_{Si}^{2}=1-\frac{0.6961663{\lambda }^{2}}{{\lambda }^{2}-{(0.0684043)}^{2}}+\frac{0.4079426{\lambda }^{2}}{{\lambda }^{2}-{(0.1162414)}^{2}}+\frac{0.897479{\lambda }^{2}}{{\lambda }^{2}-{(9.896161)}^{2}}$$

In this context, the refractive index of silica is expressed as ${n}_{Si}$, and the operating wavelength is expressed as λ in µm. The refractive index of air is assumed to be 1.

The dielectric constant of TiO_2_ is expressed by the provided equation^22^:

3 $${n}_{{TiO}_{2}}^{2}=5.913+\frac{2.441\times {10}^{7}}{({\lambda }^{2}-0.803\times {10}^{7})}$$

The excitation of surface plasmons is measured by evaluating the loss of the optical fiber. The confinement loss, quantified in decibels per centimeter (dB/cm), correlates directly with the imaginary component of the effective refractive index and is expressed mathematically by the following Eq. (4) ^22^:

4 $${\alpha }_{loss} \left[dB/cm\right]=8.686\times \frac{2\pi }{\lambda }Im({n}_{eff})\times {10}^{4}$$

where, ${k}_{0}=\frac{2\pi }{\lambda }$ is the number of waves in free space, λ is the operating wavelength, and $Im({n}_{eff})$ is the imaginary part of the effective refractive index.

Wavelength sensitivity ($Ws$) and resolution ($R$) can be defined using the equations given in references^5,22^, as shown in Eqs. (5) and (6):

5 $$Ws [nm/RIU]=\frac{\Delta {\lambda }_{peak}}{\Delta {n}_{a}}$$

where $\Delta {\lambda }_{peak}$ is the shift in the wavelength of the loss resonance peak and $\Delta {n}_{a}$ is the change in the refractive index of the analyte.

6 $$R \left[RIU\right]={\Delta n}_{a}\times \frac{{\Delta \lambda }_{min}}{\Delta {\lambda }_{peak}}=\frac{{\Delta \lambda }_{min}}{Ws}$$

In Eq. (6), $\Delta {\lambda }_{min}$ corresponds to the minimum wavelength resolution, and $\Delta {\lambda }_{peak}$ denotes the shift of the resonance peak in the wavelength domain.

The amplitude sensitivity (As) of the proposed sensor is calculated using the following formula^22^, which is defined in Eq. (7):

7 $$As \left[{RIU}^{-1}\right]=-\frac{1}{\alpha (\lambda ,{n}_{a})}\frac{\partial \alpha (\lambda ,{n}_{a})}{\partial {n}_{a}}$$

Here, $\alpha (\lambda ,{n}_{a})$ represents the loss for the given analyte with refractive index ${n}_{a}$, $\partial \alpha \left(\lambda ,{n}_{a}\right)$ is the difference between two loss spectra, and $\partial {n}_{a}$ is the change in analyte refractive index.

## Results and discussion

### Dispersion and mode field distribution

**Figure 2:**
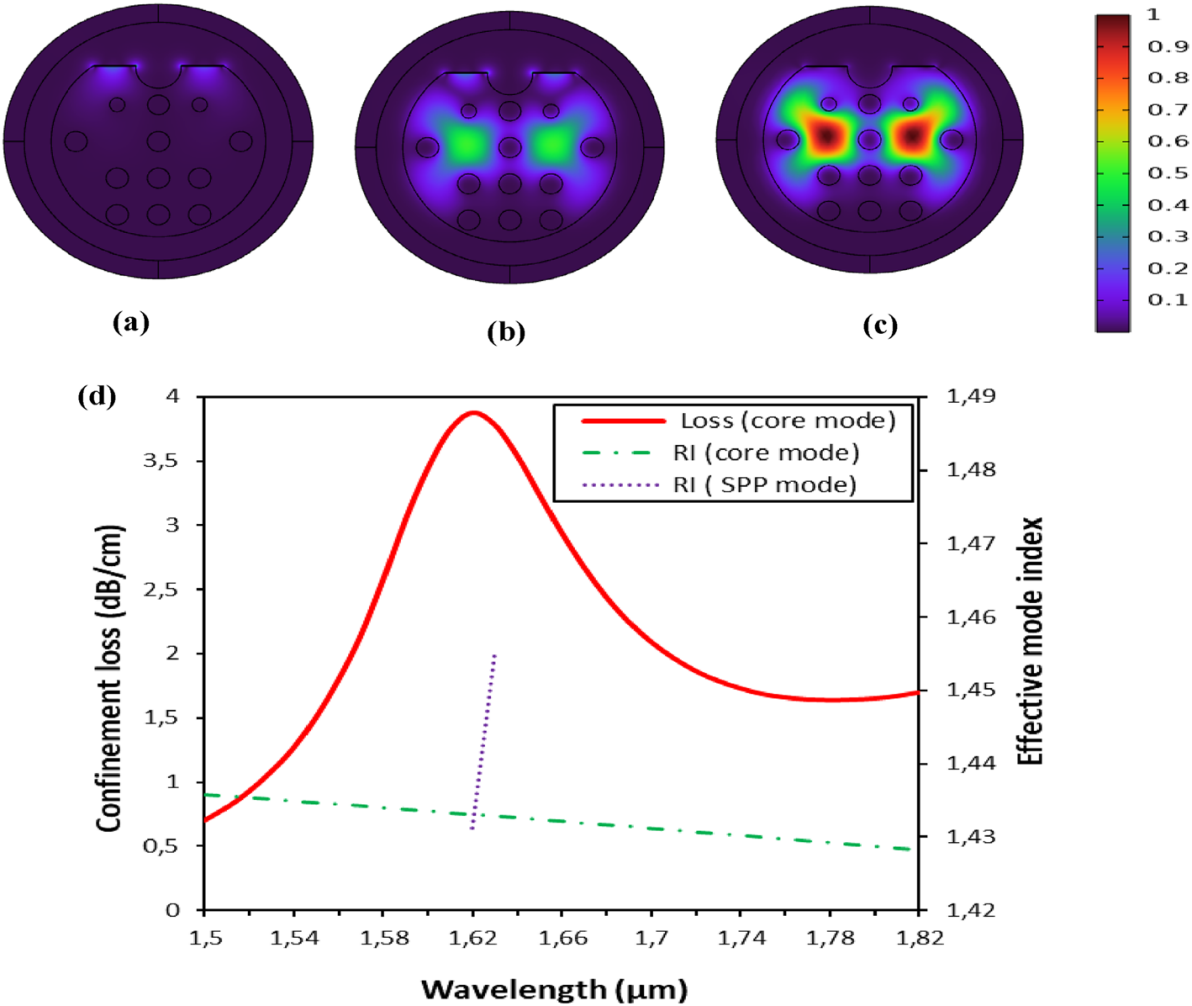
Mode field distribution of the fundamental (**a**) surface plasmon polariton (SPP) mode, (**b**) SPR mode, and (**c**) core mode at 1.65 µm. (**d**) Relationship between the dispersion of the fundamental core-guided mode and the SPP mode at n_a_ = 1.34.

Figure 2a to c visually illustrate the distribution of the mode field, providing an intuitive assessment of the coupling intensity. The color bar reflects the normalized mode field intensity distribution, ranging from 0 (indicating a weaker field) to 1 (indicating a stronger field), with the color spectrum shifting from blue to red to represent this intensity variation. Figure 2d shows the dispersion curves for the core mode and the surface plasmon polariton (SPP) mode, assuming a refractive index of 1.34. The wavelength is plotted on the x-axis and the real part of the effective refractive index, which reflects the light dispersion capabilities of the sensor, is plotted on the right y-axis. The left y-axis shows the attenuation constant per centimeter, which mirrors the pattern of the imaginary part of the effective refractive index. This measure doesn't affect the assessment of wavelength sensitivity (Ws), but effectively characterizes the light absorption or loss capabilities of the sensor.

The optimized structural parameters for the configuration are as follows: d_1_ = 1.80 µm, d_2_ = 1.00 µm, d_3_ = 1.65 µm, and pitch Λ = 3.30 µm. In addition, the silver and TiO_2_ layers have thicknesses of 65 nm and 10 nm, respectively. The aperture of the microchannel is 1.75 µm.

In this scenario, the enhanced evanescent field in the y-polarized transverse electric (TE) mode, TE^y^, is proposed to result from the excitation of a larger fraction of free electrons at the surface compared to the TE^x^ mode. The optimal power transfer becomes apparent when the phase matching condition is satisfied, facilitating the transition from the core-guided fundamental mode to the plasmonic mode. As a result, a distinct peak appears at the interface.

### Taguchi approach and ANOVA analysis

The Taguchi method is a robust optimization technique that has gained widespread recognition in various fields for its ability to systematically optimize multiple parameters and their respective levels while minimizing the need for extensive experimentation^28,30,42,43^. When applied to the task of optimizing the structural parameters of the PCF sensor, which include the diameters of the air holes (d_1_, d_2_, d_3_), the pitch (Λ), and the thickness of the silver (t_Ag_), the Taguchi method provides an efficient approach. In Table 1, we present the optimization parameters along with their associated levels. Using the Taguchi method, our goal is to identify the ideal combination of parameter settings that will increase the performance and accuracy of the PCF sensor. This methodical approach not only saves time and resources, but also fine-tunes these critical structural parameters to achieve superior results.

**Table 1:** Optimization parameters and their levels.

Parameter	Levels	
	1	2
d_1_ (µm)	1.70	1.90
d_2_ (µm)	0.80	1.20
d_3_ (µm)	1.55	1.75
$\Lambda$ (µm)	3.20	3.40
t_Ag_ (nm)	55	75

To systematically investigate the effects of these parameters, we used a Taguchi L_8_ (2^5^) orthogonal array, as shown in Table 2. In this specific design, L_8_ signifies eight experimental runs, while 2^5^ indicates five factors, each with two levels. Factors are the variables or parameters that can affect the outcome of a process or product, and levels represent the different settings or values that each factor can take. The choice of factors and their levels is crucial for conducting efficient experiments while capturing the effects of interest^28,30,42,43^. By utilizing the L_8_(2^5^) orthogonal array, researchers can systematically explore the effects of multiple factors on a process or product with a relatively small number of experiments. This structured approach not only saves time and resources but also enables the identification of optimal factor settings for improved performance or quality. In the context of Taguchi optimization, the signal-to-noise (S/N) ratio serves as a key metric. It is used to evaluate the performance of the process and to quantify the influence of different parameter combinations on the effectiveness of the sensor, specifically in terms of confinement loss (αloss). Higher confinement loss indicates strong coupling between the core and the surface plasmon polariton (SPP) mode, and vice versa. Our primary goal is to maximize the S/N ratio, which represents an optimal balance between desired performance (signal) and unwanted variation (noise), ultimately resulting in an improved PCF sensor. The signal-to-noise (S/N) ratios were determined using the next criterion according to Eq. (8)^30^:

8 $$\mathrm{Larger \, is \, better}:{(S/N)}_{i}=-10{{\text{log}}}_{10}\left(\frac{1}{n}\sum_{i=1}^{n}\left(\frac{1}{{Y}_{i}^{2}}\right)\right)$$

where *n* is the number of simulation tests performed and ${Y}_{i}$ is the measured response (confinement loss) for the ith simulation. Table 2 shows the numerical results for the PCF-SPR sensor's confinement loss peak ${\alpha }_{loss}^{peak}$, wavelength peak ${\lambda }_{peak}$, and the corresponding signal-to-noise (S/N) ratios obtained by the L_8_ experimental layout.

**Table 2:** The Taguchi L_8_(2^5^) orthogonal table.

Experiment Test	Factors levels					Outputs		
	d_1_	d_2_	d_3_	$\Lambda$	t_Ag_	${\alpha }_{loss}^{peak}$(dB/cm)	${\lambda }_{peak}$(µm)	$S/N$(dB)
1	1.7	0.8	1.55	3.2	55	14.0790	1.8	22.9714
2	1.7	0.8	1.55	3.4	75	4.1233	1.76	12.3049
3	1.7	1.2	1.75	3.2	55	23.8960	1.84	27.5665
4	1.7	1.2	1.75	3.4	75	7.0851	1.8	17.0069
5	1.9	0.8	1.75	3.2	75	25.0240	1.8	27.9671
6	1.9	0.8	1.75	3.4	55	29.1150	1.96	29.2823
7	1.9	1.2	1.55	3.2	75	5.9560	1.68	15.4991
8	1.9	1.2	1.55	3.4	55	5.5555	1.82	14.8945

**Figure 3:**
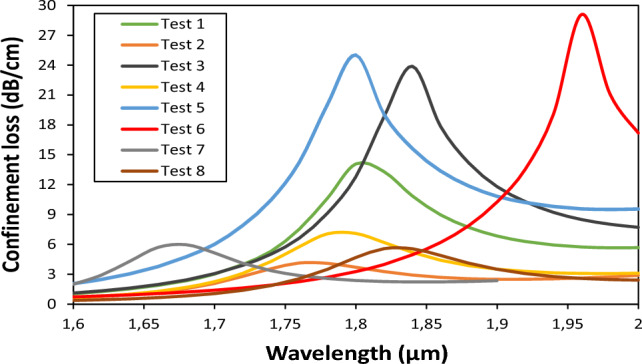
Loss curves for the eight Taguchi tests.

Figure 3 shows the loss curves corresponding to all the experimental tests described in Table 2**.**

To assess the impact of each key parameter, it is critical to calculate the mean signal-to-noise (S/N) responses for each corresponding level. This is done by summing the results associated with each level from the orthogonal table and dividing this sum by the number of tests performed at that level. The significance of each factor can then be determined by calculating the difference between the maximum and minimum mean S/N ratios across the two levels, referred to as the delta, as shown in Table 3. A larger difference indicates a greater effect of that control factor. Examination of the response data presented in Table 3 indicates that the Air Hole Diameter (d_1_) factor has the most significant influence.

**Table 3:** Response Table for Signal to Noise Ratios.

Level	d_1_	d_2_	d_3_	$\Lambda$	t_Ag_
1	19.96	23.13	16.42	23.50	23.68
2	21.91	18.74	25.46	18.37	18.19
Delta	1.95	4.39	9.04	5.13	5.48
Rank	5	4	1	3	2

**Figure 4:**
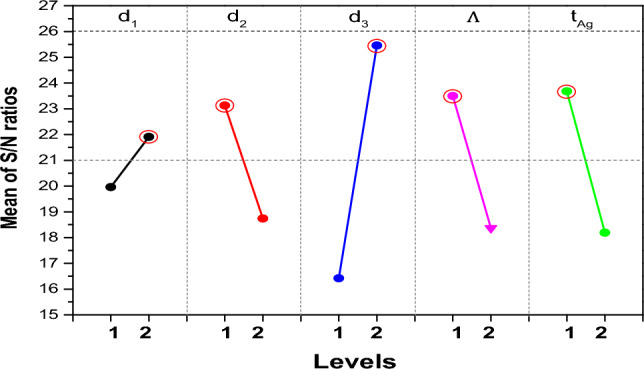
Main effects plot for S/N ratios.

In Fig. 4, the plot of signal to noise (S/N) versus each key parameter shows that the maximum confinement loss occurs when d_1_ is at level 2, d_2_ is at level 1, d_3_ is at level 2, Λ is at level 1, and t_Ag_ is at level. It is noteworthy that the optimal combination obtained (d_1_ = 1.9 µm, d_2_ = 0.8 µm, d_3_ = 1.75 µm, Λ = 3.2 µm, and t_Ag_ = 55 nm) was not initially included in the L_8_ orthogonal array provided by Taguchi's method. It is noteworthy that the simulation of the confinement loss using these optimal parameters yields a value of 31.536 dB/cm, exceeding the values obtained in all other tests performed.

**Figure 5:**
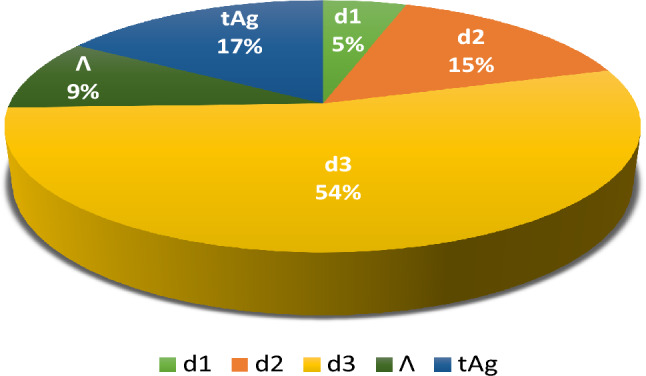
Contributions of key parameters to the confinement loss of the PCF sensor.

The analysis of variance (ANOVA) framework, as applied in the L_8_ Taguchi approach presented in Table 4, is used to determine the percentage contribution of each significant parameter to the increase in confinement loss. DF is the degree of freedom associated with each factor, Seq-SS is the sequential sum of squares, and Adj-MS is the adjusted sum of squares divided by the degrees of freedom. Table 4 and Fig. 5 together show that the most significant contributions are associated with the parameter d_3_, which accounts for 53%, and t_Ag_, which contributes 16%. Conversely, parameters Λ and d_1_ show minimal contributions of 9% and 5%, respectively, to α_loss_. Also the factor d_3_ seems to have a statistically significant impact on the variability of the data, as indicated by its low p-value (typically less than 0.05). The F-values and percentage contributions show the relative importance of each parameter.

**Table 4:** Results of the ANOVA on the sensor confinement loss.

Source	DF	Seq-SS	Adj-MS	Contribution	F-value	P-value
d_1_	1	33.90	33.896	4.65%	4.04	0.182
d_2_	1	111.37	111.368	15.29%	13.26	0.068
d_3_	1	383.73	383.732	**52.69%**	**45.70**	**0.021**
$\Lambda$	1	66.56	66.563	9.14%	7.93	0.106
t_Ag_	1	115.95	115.954	15.92%	13.81	0.065
Error	2	16.79	8.396	2.31%		
Total	7	728.31		100.00%		

Significant values are in bold.

### Multiple linear regression model

Multiple linear regression (MLR) analysis is a statistical modeling method used to examine the correlation between a dependent variable (in this case, response) and two or more independent variables (designed as inputs). Using the data extracted from the Taguchi table, we can create an MLR model to examine how the five control factors (d_1_, d_3_, d_3_, $\Lambda$, t_Ag_) relate to the confinement loss ${\alpha }_{Loss}$. The regression analysis is performed using Matlab software. The explicit equation for this model is given below:

9 $${\boldsymbol{\alpha }}_{{\varvec{L}}{\varvec{o}}{\varvec{s}}{\varvec{s}}}\left(dB/cm\right)=14.35+2.06{\times {\varvec{d}}}_{1}-3.73{\times {\varvec{d}}}_{2}+6.93{\times {\varvec{d}}}_{3}-2.88\times{\varvec{\Lambda}}-3.81{\times {\varvec{t}}}_{{\varvec{A}}{\varvec{g}}}$$

We specify that for the above MLR equation, the key parameters are encoded (-1 for the low level and + 1 for the high level).

Based on the predicted results of the MLR model presented in Table 5, a high R-squared (R^2^) value of approximately 97.69% in the variance of the dependent variable suggests that the model is effective in capturing the variation in the data. The adjusted R-squared (${R}_{Adj}^{2}$) takes into account the number of predictors in the model and penalizes the R-squared for including irrelevant predictors. With an ${R}_{Adj}^{2}$ value of 91.93%, we see that approximately 91.93% of the variance is explained, taking into account the influence of the number of predictors. This provides a more conservative estimate of the explanatory power of the model, especially in cases with multiple predictors such as ours. The predictive ability of the model for new data points is assessed by the predicted R-squared (${R}_{pred}^{2}$). Its value of 63.11% indicates that the model can predict approximately 63.11% of the variance in new observations.

**Table 5:** Comparaison of simulated and prediced MLR values.

Tests	d_1_	d_2_	d_3_	$\Lambda$	t_Ag_	Simulated values	Predicted values
1	1.7	0.8	1.55	3.2	55	14.0790	15.7928
2	1.7	0.8	1.55	3.4	75	4.1233	2.4095
3	1.7	1.2	1.75	3.2	55	23.8960	22.1822
4	1.7	1.2	1.75	3.4	75	7.0851	8.7989
5	1.9	0.8	1.75	3.2	75	25.0240	26.1469
6	1.9	0.8	1.75	3.4	55	29.1150	27.9921
7	1.9	1.2	1.55	3.2	75	5.9560	4.8331
8	1.9	1.2	1.55	3.4	55	5.5555	6.6784

Under ideal operating conditions (optimal conditions), the MLR model predicts the optimal value of the confinement loss value (${\widehat{\alpha }}_{Loss}$) to be 33.761 dB/cm. Performing the FEM simulation with these optimized settings yields an observed value (${\alpha }_{Loss}$) of 31.536 dB/cm, with a relative error of :$\left|\frac{{\alpha }_{Loss}-{\widehat{\alpha }}_{Loss}}{{\alpha }_{Loss}}\right|\times 100\approx 7\%$. This level of error is considered acceptable in engineering.

### Confinement loss with changing analyte RI (n_a_)

**Figure 6:**
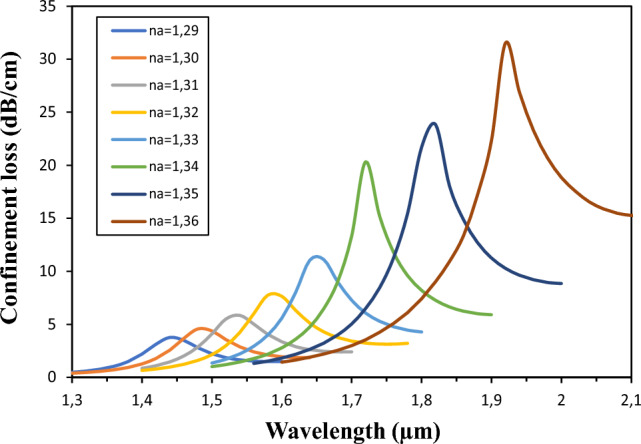
Confinement loss for varying analyte RI from a 1.29 to 1.36; using optimal sensor parameters.

The loss peak approach is widely accepted for evaluating the efficiency of an SPR sensor. Increased losses contribute to an expanded evanescent field within the cladding of the photonic crystal fiber (PCF), thereby increasing sensitivity. The proposed sensor exhibits increased sensitivity, capable of detecting even subtle variations in the refractive index (RI) of the analyte. This is particularly evident when the effective RI (n_eff_) of the fundamental mode is significantly affected by the analyte RI (n_a_), as shown in the confinement loss spectra in Fig. 6. In this particular scenario, a noticeable shift in the resonance wavelength accompanies a change in analyte RI from 1.29 to 1.36. It can be seen that as the analyte RI increases, the confinement loss also increases, causing the resonance peak to shift to higher values. This phenomenon is due to the fact that variations in RI induce changes in both the propagation constant and the kinetic binding energy^44^. Consequently, the confinement loss exhibits a minimum of 3.7654 dB/cm at 1.44 μm with n_a_ value of approximately 1.29, while the maximum confinement loss peak of 31.536 dB/cm is observed at 1.92 μm with n_a_ value of approximately 1.36.

### Wavelength sensitivity

In the general context, the wavelength interrogation method is used to determine the wavelength sensitivity (Ws), which is defined by Eq. (5). In our proposed SPR sensor, we observed Δλ_peak_ values of 40, 60, 40, 80, 60, 100, 100 nm as n_a_ varied from 1.29 to 1.30, 1.30 to 1.31, 1.31 to 1.32, 1.32 to 1.33, 1.33 to 1.34, 1.34 to 1.35, and 1.35 to 1.36, respectively. Accordingly, the maximum Ws values obtained were 4000, 6000, 4000, 8000, 6000, 10,000, and 10,000 nm/RIU. Consequently, the wavelength sensitivity reaches a peak value of approximately 10,000 nm/RIU within the analyte RI range of 1.34 to 1.36.

### Amplitude sensitivity

**Figure 7:**
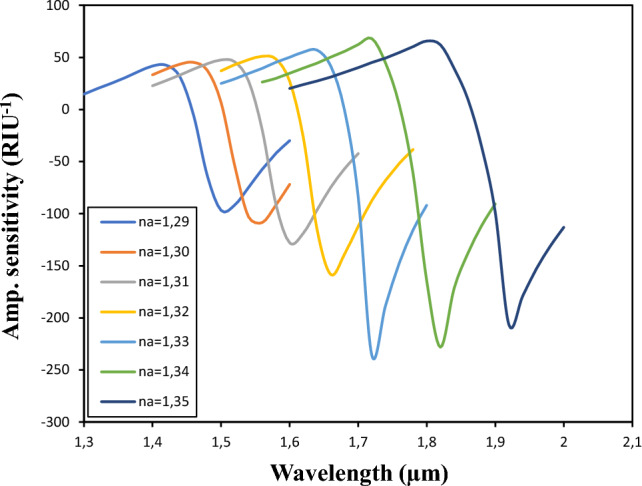
Amplitude of sensitivity as the refractive index of the analyte varies from 1.29 to 1.35.

Unlike wavelength sensitivity, amplitude sensitivity provides a simple and inexpensive method of measuring sensitivity at a specific wavelength. The amplitude sensitivity observed by varying the sample refractive index (RI) from 1.29 to 1.36 is shown in Fig. 7. As shown in the figure, the amplitude sensitivity shows an increase as the sample RI increases from 1.29 to 1.33. The peak shifts to a higher wavelength, indicating an enhanced interaction between the evanescent field and the surface plasmon polariton (SPP) mode. Consequently, the amplitude sensitivity reaches a maximum value of approximately 235.882 RIU^−1^ at n_a_ = 1.33 and an operating wavelength of 1.72 μm.

### Machine learning models

Machine learning models were used to optimize and predict the confinement loss of the PCF sensor using simulation data. The input factors were air hole diameters (d_1_, d_2_, d_3_), pitch (Λ), and silver thickness (t_Ag_). Their effects on the efficiency of PCF sensors were evaluated by a full experimental design (2^5^) with 32 samples, since machine learning models require a large dataset. The dataset and model architecture were used to evaluate the effectiveness of two different Artificial Neural Network (ANN) optimization techniques, namely Multi-Layer Perceptron (MLP) and Particle Swarm Optimization (PSO).

### Statistical error analysis

The following coefficients^45^ were calculated to monitor the performance of the models used in this analysis: MLR-ANN and PSO-ANN

10 $$VAF=\left[1-\frac{var\left(y-\widehat{y}\right)}{var\left({y}_{i}\right)}\right]\times 100$$

11 $$RMSE=\sqrt{\frac{1}{N}\sum_{i=1}^{N}{\left({y}_{i}-\widehat{{y}_{i}}\right)}^{2}}$$

12 $$MAPE=\frac{1}{N}\sum_{i=1}^{N}\left|\frac{{y}_{i}-\widehat{{y}_{i}}}{{y}_{i}}\right|\times 100$$

13 $${R}^{2}=1-\frac{\sum_{i=1}^{N}{\left({y}_{i}-\widehat{{y}_{i}}\right)}^{2}}{\sum_{i=1}^{N}{\left(\overline{y }-{y}_{i}\right)}^{2}}$$

14 $${R}_{Adj}^{2}=1-\left(\left(1-{R}^{2}\right)\frac{N-1}{N-k-1}\right)$$

Here, N is the number of samples, VAF is the variance accounted for, RMSE is the root mean square error, MAPE is the mean absolute percentage error, ${R}^{2}$ is the coefficient of determination, and ${R}_{Adj}^{2}$ is the adjusted ${R}^{2}$. Here, ${y}_{i}$ is the actual value, $\widehat{{{\text{y}}}_{{\text{i}}}}$ is the predicted value, $\overline{{\text{y}} }$ is the average value of y, and *k* is the number of features (input variables).

### MLP-ANN optimization

**Figure 8:**
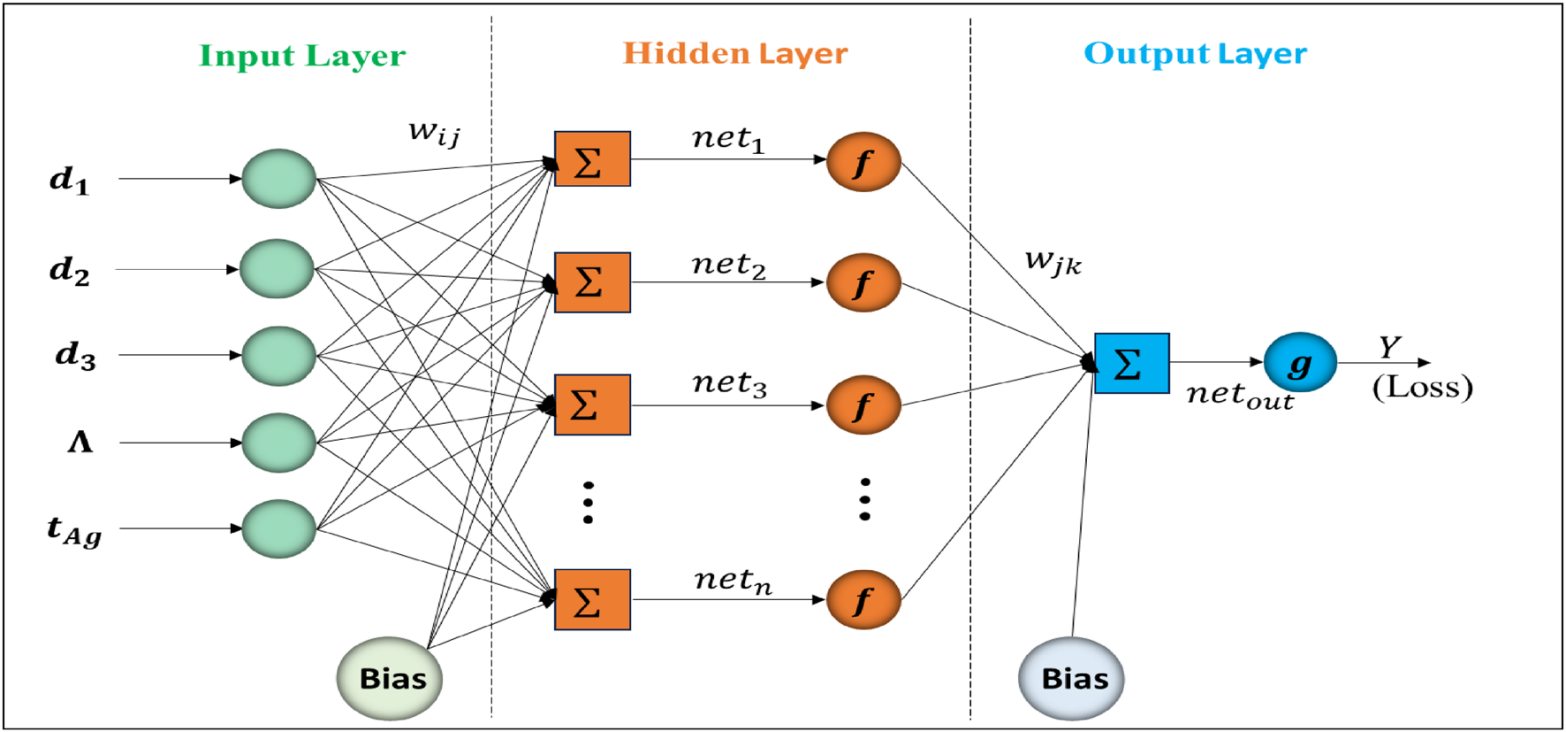
The MLP-ANN Structure.

A Multi-Layer Perceptron Artificial Neural Network (MLP-ANN) is a type of artificial neural network that uses the Multi-Layer Perceptron architecture. This architecture is characterized by its composition of multiple connected layers of neurons, which typically include an input layer, one or more hidden layers, and an output layer. ANNs are widely used in the field of machine learning, where they are applied to various tasks such as classification, regression, and pattern recognition. Figure 8 provides a visual representation of the network structure of the MLP used in this study.

The overall network consists of multiple interconnected layers, and learning is accomplished by adjusting weights and biases during the training phase, typically using optimization techniques such as gradient descent. The following equation is a mathematical representation of the feedforward process in a neural network. It calculates the output of a given neuron in the output layer based on the inputs, weights, and biases from the previous layer, incorporating activation functions:

15 $${Y}_{k}={\varvec{g}}\left({\sum }_{j=1}^{q}{k}_{j}^{0}{\varvec{f}}\left(\left({\sum }_{i=1}^{p}{w}_{ij}{x}_{i}\right)+{b}_{j}^{h}\right)+{b}^{0}\right)$$

In this equation, ${Y}_{k}$ represents the output of neuron k within the neural network, $g$ and $f$ denote the activation function of neurons in the output layer and hidden layers respectively (Fig. 8), $q$ represents for the number of neurons in the previous layer and, ${w}_{ij}$ represents the weight of the connection between neuron *i* in the input layer and neuron j in the hidden layer. ${b}_{j}^{h}$ represents the bias term associated with neuron j in the hidden layer and ${b}^{0}$ represents the bias term associated with neuron *k* in the output layer. Several networks with different numbers of hidden layer neurons were trained and then evaluated. The architecture of the ANN used in this investigation is characterized by a feed-forward structure using sigmoid activation functions within the hidden layers and a linear activation function at the output node.

**Figure 9:**
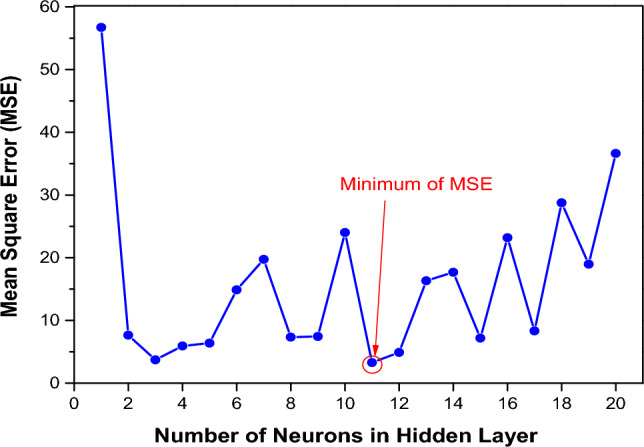
Mean square error between measured data and model output from variation with the number of neurons in the hidden layer.

Following Bishop's seminal work in 1995^46^, which suggests that more than one hidden layer is often unnecessary, our architectures have only one hidden layer. A back-propagation gradient descent algorithm was used to train the ANN. The dataset was carefully divided into three distinct subsets for the duration of the training phase: a training dataset (70%), a test dataset (15%), and a validation dataset (15%). The number of neurons in the hidden layer was systematically adjusted in the range of 1 to 20 in order to evaluate the performance of the model. The mean squared error between the simulation data and the model output, shown in Fig. 9, was expressed as a function of the number of neurons in the hidden layer. The selection of the most effective network depended on its ability to predict responses with the lowest mean squared error. Consequently, as shown in Fig. 9, our results showed that the optimal network configuration was achieved with a 5:11:1 structure (11 neurons in the hidden layer). This result is consistent with the formula derived from the literature^47^:

16 $${N}_{neurone}=\frac{{N}_{in}+\sqrt{{N}_{p}}}{L}$$

In this equation, L is the number of hidden layers (in our case, L = 1), ${N}_{in}$ is the number of inputs (in our case, ${N}_{in}=5$), and ${N}_{p}$ is the number of samples (in our case, ${N}_{p}$=32).

The architecture, parameters and optimization process of the ANN network are shown in Table 6**.**

**Table 6:** Architecture and parameters of ANN.

Aspect	Description
Input layer	5 neurons
Hidden layers	11 neurons
Activation function	Sigmoid
Output layer	1 neuron
Learning rate	0.5
Number of epochs	500
Loss function	MSE
Momentum constant	0.9

**Figure 10:**
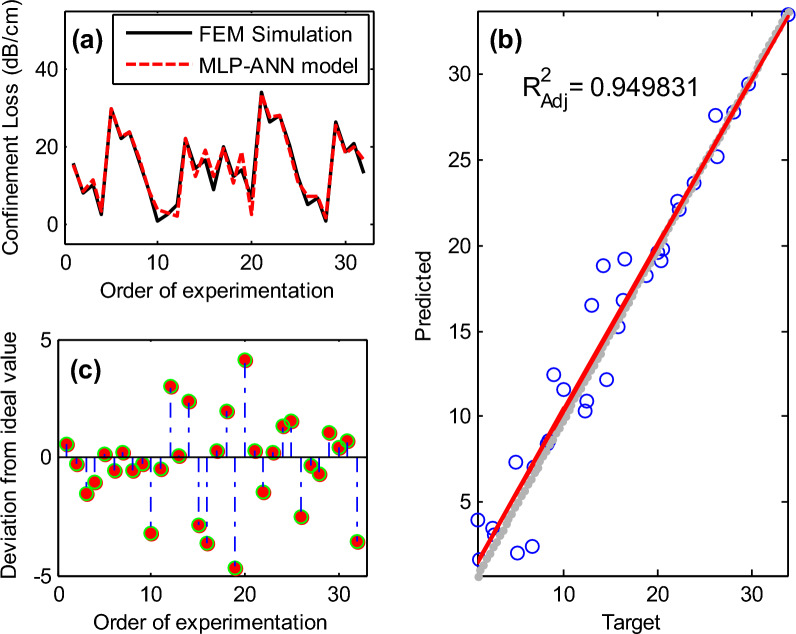
(**a**) comparison of observed and predicted confinement loss using MLP-ANN model; (**b**) statistical analysis fit of the MPL-ANN model; (**c**) Deviation analysis of MLP- ANN model predictions from actual confinement loss values.

Figure 10 presents a comprehensive assessment of the MLP-ANN model's performance in predicting PCF sensor efficiency: (a) Comparison of observed (FEM simulation) and predicted data using the MLP-ANN model. It serves as a visual representation of how well the model's predictions match the actual data, providing insight into the model's predictive accuracy. (b) Statistical Analysis Fit of the MLP-ANN Model. It provides insight into the goodness of fit of the model by assessing how well the predicted values match the actual data. In addition, subplot (c) evaluates the deviation of the predicted values generated by the MLP-ANN model from the actual values. This provides a clear understanding of the model's prediction errors and any discrepancies between the predicted and observed data points, facilitating further analysis and refinement of the model if necessary.

The achieved correlation coefficient of 0.949, which is close to 1, indicates a strong correlation. Furthermore, the values for RMSE, VAF, MAPE, ${R}^{2}$, and ${R}_{adj}^{2}$ underscore the robust predictive capability of the MLP-ANN model.

### PSO-ANN optimization

**Figure 11:**
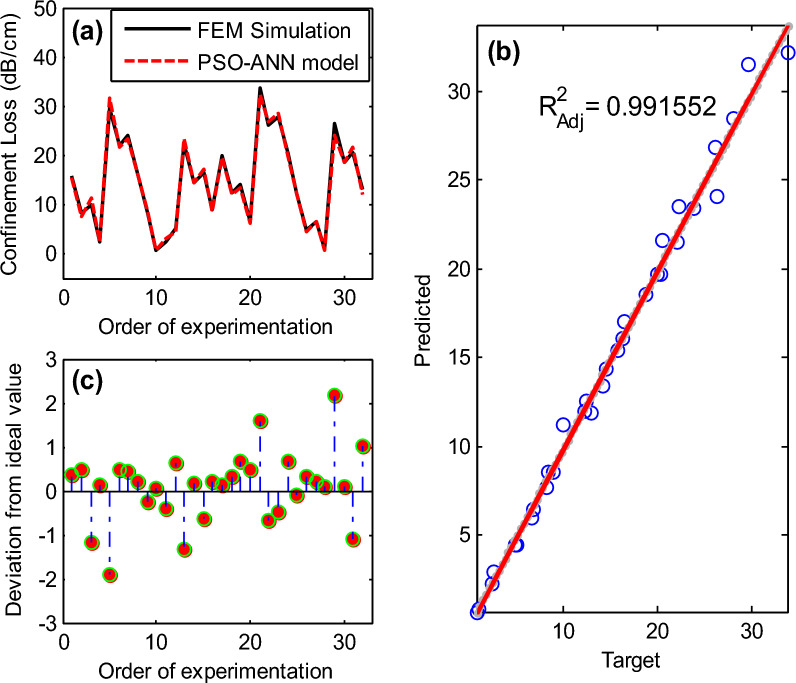
(**a**) Comparison of observed confinement loss values using the PSO-ANN model; (**b**) statistical analysis fit of the PSO-ANN model; (**c**) analysis of the deviation of the PSO-ANN model prediction from the actual confinement loss values.

Using the Particle Swarm Optimization (PSO) algorithm in conjunction with a traditional Artificial Neural Network (ANN) is a promising approach^48^. As mentioned earlier, in a typical ANN structure, there are several layers: an input layer, one or more hidden layers, and an output layer. These layers are connected by a network of weighted connections, where the value of each neuron is determined by the sum of the connections within the neuron, weighted by their respective values. An activation function, typically sigmoidal in nature, is then applied to this value. The weights of the network are traditionally tuned by error backpropagation and gradient descent. Incorporating the PSO algorithm into this framework helps optimize the connection weights within the ANN, with the goal of identifying the optimal weight values that produce the best results. Initially, the PSO algorithm generates a population of particles, each of which is used within the neural network. The fitness of each particle, representing a potential solution set, is evaluated, and pertinent local and global information is retained within each particle. PSO then uses this information to update particle velocities and effectively explore the solution space. The PSO-ANN model configured with a swarm size of 150, a cognitive coefficient C_1_ of 1.5, a social coefficient C_2_ of 2, and an inertia weight W of 0.9 provides the most accurate prediction results, resulting in an exceptionally high regression coefficient of approximately 0.99 (as shown in Fig. 11). The global parameters of the PSO algorithm for the optimization of the ANN network are shown in Table 7.

**Table 7:** Parameters of PSO algorithm.

Parameter	Description
Number of particles	176
Fitness function	MSE
Inertia weight	0.65
Cognitive learning factor	0.5
Social learning factor	2
Maximum velocity	5

The performance metrics, including RMSE, VAF, MAPE, and R^2^ (detailed in Table 8), consistently show that the PSO-ANN model outperforms the MLP-ANN model in terms of prediction accuracy. In particular, the RMSE obtained at the 500th iteration was exceptionally low.

**Table 8:** Key performance metrics for MLP-ANN and PSO-ANN models.

Model	MAPE (%)	VAF (%)	RMSE	${R}^{2}$	${R}_{Adj}^{2}$
MLP-ANN	0.31	95.25	1.95	0.95	0.94
PSO-ANN	0.05	99.19	0.8	0.99	0.99

### Comparative study

**Figure 12:**
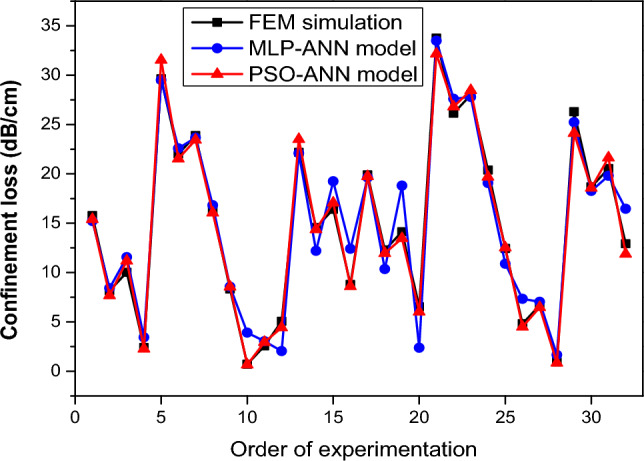
Comparison plot of simulation data for PCF sensor confinement loss versus predicted data obtained by MLP-ANN and MLP-ANN models.

In this section, we conducted a comparative analysis between the results generated by two different learning machine models used to predict the confinement loss of the PCF-SPR sensor and the results obtained through by finite element simulations. Figure 12 and Table 8 provide a visual and numerical representation of this comparative study. Figure 12 visually represents the discrepancies between the predicted values of the MLP-ANN and PSO-ANN models with respect to the actual data values for the confinement loss. It is evident that the PSO-ANN model provided an efficient prediction that was more reliable than the MLP-ANN model.

Furthermore, when examining the key performance metrics presented in Table 8, it is evident that the PSO-ANN model outperforms the MLP-ANN model. Specifically, metrics such as R^2^ (coefficient of determination), RMSE (root mean square error), VAF (variance accounted for), and MAPE (mean absolute percentage error) all indicate superior performance for the PSO-ANN model. Taken together, these results indicate that the PSO-ANN model excels in predicting the optical properties of the PCF-SPR sensor.

### Genetic algorithm optimization

To maximize the sensor confinement loss, the independent parameters [air hole diameters (d_1_, d_2_, d_3_), pitch (Λ) and silver thickness (t_Ag_)] were also optimized by genetic algorithm (GA). The optimized PSO-ANN model was used as the objective function of the GA. The optimization was performed under constraints to obtain optimal conditions predicted in the experimental range. The experimental ranges adopted in the Taguchi design were used as bounds for the five input variables. The optimization problem to be solved by the GA was constructed as follows:

17 $$Maximize \, objective \, function \, (optimized \, PSO-ANN)\left\{\begin{array}{c}{1.7\mu m\le d}_{1}\le 1.9\mu m\\ {0.8\mu m\le d}_{2}\le 1.2\mu m\\ {1.55\mu m\le d}_{3}\le 1.75\mu m\\ 3.2\mu m\le \Lambda \le 3.4\mu m\\ 55nm\le {t}_{Ag}\le 75nm\end{array}\right.$$

The optimization process was continued until very low mean sum of square error (MSE) and root mean square error (RSME) values were obtained between the mean and individual fitness values. After mutation, the optimization cycle resumed, and if the target result was not achieved, the whole population was used for the next cycle of breeding, crossover, and mutation. The objective function was written as a MATLAB file using the PSO-ANN model. The GA parameters used for optimization are shown in Table 9.

**Table 9:** GA parameters used for optimization.

Option GA	Value
Population size	100
Cross fraction	0.8
Migration fraction	0.2
Size of selection function	2

**Figure 13:**
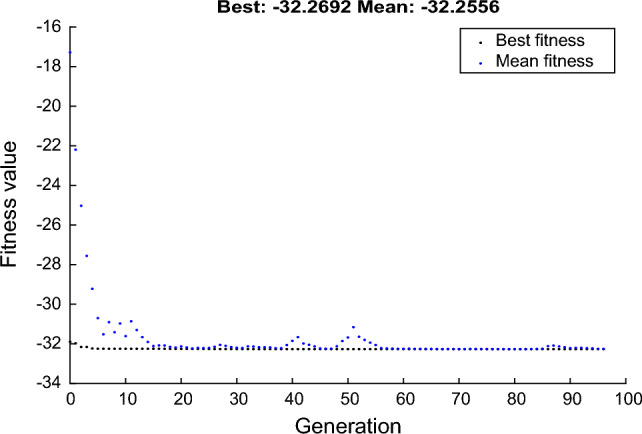
Variation of fitness value as a function of generations during genetic algorithm optimization.

The evolution of the fitness value as a function of the number of generations is shown in Fig. 13. It is clear from this figure that from the 60th generation, the fitness value remains constant with an average value of − 32.2692. The optimization performed by the GA resulted in the following conditions: d_1_ = 1.9 µm, d_2_ = 0.8 µm, d_3_ = 1.75 µm, Λ = 3.27 µm and t_Ag_ = 58.02 nm. Under these optimized conditions, the predicted value of the confnement loss is 32.2692 dB/cm.

## Conclusion

In this study, we used the Taguchi optimization approach to efficiently identify optimal structural parameters for a PCF-SPR sensor, including air hole diameters (d_1_, d_2_, d_3_), pitch (Λ), and silver layer thickness (t_Ag_). Then, we developed MLP-ANN and PSO-ANN machine learning models to predict the confinement loss based on these parameters. The results showed that the PSO-ANN model outperformed the MLP-ANN model, achieving an impressive R^2^ value of 0.99, indicating exceptional prediction accuracy. Taguchi optimization demonstrated its effectiveness in minimizing the number of trials required for sensor optimization. Finally, a genetic algorithm (GA) was applied to further optimize the sensor conditions with the goal of increasing the confinement loss. Under the optimized parameters (d_1_ = 1.9 µm, d_2_ = 0.8 µm, d_3_ = 1.75 µm, Λ = 3.27µm, t_Ag_ = 58.02 nm), the GA approach yielded a maximum confinement loss of 32.2692dB/cm. These combined results underscore the comprehensive optimization approach using Taguchi optimization, machine learning models, and genetic algorithms for improved performance in PCF-SPR sensor design.
